# Antimicrobial Susceptibility and Clinical Findings of Anaerobic Bacteria

**DOI:** 10.3390/antibiotics11030351

**Published:** 2022-03-07

**Authors:** Fernando Cobo

**Affiliations:** Department of Microbiology, Instituto de Investigación Biosanitaria Ibs.GRANADA, University Hospital Virgen de las Nieves, 18014 Granada, Spain; fernando.cobo.sspa@juntadeandalucia.es

Anaerobic microorganisms are the most abundant components of the normal human microbiota; they colonize mucous membranes such as the oral cavity and the gastrointestinal and female genital tracts, and they are common pathogens in human populations. The presence of anaerobic microorganisms predominates in several clinical syndromes, and this fact could be attributed to the large numbers of these bacteria that reside on mucous membranes, the production of a wide variety of virulence factors, their synergy with other aerobic bacteria, and the increased resistance of these microorganisms to some antimicrobials. However, most clinically significant anaerobes are involved in mixed infections alongside aerobic bacteria.

In some circumstances, especially after the breakdown of mucosal barriers, these bacteria can spread from indigenous microbiota into normally sterile body sites, and they may be responsible for severe diseases such as bloodstream infections.

Infections due to anaerobic isolates may sometimes be missed because of the special measures required for their transportation. Other critical factors regarding the successful isolation of these microorganisms in the microbiology laboratory include incubation in anaerobic atmosphere, the use of specialized culture media, and prolonged culture [1]. Moreover, several infections caused by anaerobes are important because in recent years, higher resistance rates of these microorganisms to some antimicrobial agents have been observed [2]. The combination of all of these factors means it is crucial to know when an anaerobic infection is vital in order to use appropriate microbiologic methods to identify the bacteria and to select the correct treatment.

Antibiotic resistance among anaerobic microorganisms has increased significantly in recent decades [3,4]). However, resistance rates vary widely among different geographic regions. Regarding the *Bacteroides fragilis* group, resistance to penicillin may be observed in around 80–90% of isolates, while a higher proportion of the strains (20%) are also resistant to amoxicillin–clavulanate. The overall resistance rate to carbapenems is very low (<1%), although some studies have reported higher rates [5]. In recent years, the most significant changes in *Bacteroides* spp. have been the increase in the resistance rate to clindamycin, ranging from 30 to 50%. The most active drug against *Bacteroides* spp. is metronidazole, and resistance to this antibiotic remains rare, although reports are emerging worldwide [6,7].

Regarding *Prevotella*, resistance rate to penicillin is also increasing [7], and their resistance rate to clindamycin ranges from 11 to 40%. Overall, no resistance to metronidazole was found in *Prevotella* isolates, although in some studies, this resistance has been detected [8,9]. On the other hand, resistance to antibiotics has been found in very few isolates of *Fusobacterium*, except for penicillin due to b-lactamase production.

Among *Clostridium*, *C. difficile* shows a high resistance rate to imipenem (90%) and clindamycin (40%) but no resistance to metronidazole or vancomycin. Among other *Clostridium* species, resistance may be observed for all antimicrobials, except for imipenem. Non-spore-forming, Gram-positive bacilli are intrinsically resistant to metronidazole, but they are highly susceptible to penicillin, b-lactams/blactamase inhibitor, and carbapenems. The rate of resistance to clindamycin is highest for *Actinomyces* and lowest for *Cutibacterium*.

Finally, regarding GPACs, no resistance to carbapenems and b-lactams/blactamase inhibitor can be observed in any genera.

On the other hand, resistance to moxifloxacin is high in all genera, ranging between 25% and 46%, but the level of resistance depends on the breakpoints chosen [10]. In this group of microorganisms, the resistance rate to clindamycin is much higher for *Finegoldia* (around 50%) and *Peptoniphilus* (around 40%). Some GPAC strains have shown resistance to metronidazole [11].

In conclusion, routine antimicrobial susceptibility testing for anaerobes provides information regarding the global situation and permits empirical therapies to be selected in accordance with local data on resistant strains.

This Special Issue includes seven full research articles and one case report focused on clinical features and antimicrobial susceptibility to anaerobic microorganisms.

Among the full research articles included, Dr. Hanane Zerrouki et al. reported the prevalence and antimicrobial resistance of *Paeniclostridium sordellii* in hospital settings in Algeria [12]. Surface samples were collected from intensive care units (ICUs) and surgical wards. Identification was performed using MALDI-TOF MS and minimum inhibitory concentrations (MICs); several antimicrobial agents and biocides were analyzed. Ninety isolates of *P. sordellii* were obtained, and no lethal and hemorrhagic toxin genes were detected. This pathogen was susceptible to all antibiotics tested, although these strains exhibited resistance to the tested biocides.

On the other hand, Dr. Florian Baquer et al. compared two broth microdilution kits and one gradient diffusion strip using the reference agar dilution method with regard to *Clostridiales* isolates [13]. The results varied greatly according to the antibiotic, but vancomycin resulted in agreement above 90% for the three methods. The highest rate of error was observed for clindamycin.

Dr. Sylvia Valdezate et al. [14] examined the genomes, taxonomy, and phylogenetic relationships regarding other *B. fragilis* genomes of two *B. fragilis* strains resistant to meropenem + EDTA and other antimicrobial drugs. Both strains possessed *cfi*A genes (*cfi*A14b and the new *cfi*A28) along with other antimicrobial resistance mechanisms. The surveillance of resistance mechanisms in these and other strains was strongly recommended by the authors.

Dr. Jens Strohäker et al. [15,16] published two papers in this Special Issue; the first report was focused on the prevalence and clinical significance of anaerobic bacteria in major liver resection. The main objective of this study was to analyze the prevalence and outcome of anaerobic infections in major hepatectomies. They described 245 major hepatectomies, and only 13 patients suffered from anaerobic infections, so they concluded that anaerobic infections in liver resections are uncommon in these types of patients. The second paper was focused on the clinical presentation and incidence of anaerobic bacteria in surgically treated biliary tract infections and cholecystitis. The authors analyzed bacterial cultures from biliary samples, and from 365 patients with culture testing, anaerobic bacteria grew in only 42; older patients with hepatic abscesses and gallbladder perforation were particularly susceptible.

Moreover, Dr. Fernando Cobo et al. [17,18] also published two papers; the first article described the clinical and microbiological characteristics of breast abscesses caused by anaerobic microorganisms. From these lesions, 35 clinically significant anaerobic bacteria were isolated. The most frequent anaerobes were *Finegoldia magna* and *Actinomyces* spp. High overall resistance rates to clindamycin, metronidazole, and moxifloxacin were detected in some anaerobes. The second study presented clinical findings and the antimicrobial susceptibility of anaerobic microorganisms isolated from bloodstream infections. A total of 141 clinically significant isolates were analyzed. The most frequent anaerobes were *Bacteroides* spp, *Clostridium* spp, and Gram-positive anaerobic cocci (GPACs). High overall resistance rates to clindamycin and penicillin were observed in GPACs and *Bacteroide*s; in comparison, low resistance rates to almost all antimicrobials were observed for *Clostridium*.

Finally, Dr. Charlotte Kaeuffer et al. [19] described the first case report of bacteremia caused by multidrug-resistant *Bacteroides faecis*, which produced carbapanemase encoded by the *bla*_Cfi_ gene. This report highlighted the importance of knowledge of anaerobic bacteria for a systematic antimicrobial stewardship procedure following bacteremia.

This Special Issue includes multidisciplinary research focused on clinical aspects and the antimicrobial susceptibility of clinically significant anaerobic microorganisms. These contributions suppose an improvement in both the knowledge of these infections and the antimicrobial treatment.
